# Supplementary material for: Spaceflight induces changes in gene expression profiles linked to insulin and estrogen
Source: Commun Biol. 2024 Jun 11;7:692. doi: 10.1038/s42003-023-05213-2 (PMC11166981; doi:10.1038/s42003-023-05213-2)
Supplement: Supplementary file 2 — Supplemental Material [file 42003_2023_5213_MOESM2_ESM.pdf]

Supplemental Information

Spaceflight induces changes in gene expression profiles linked to insulin and estrogen

Supplementary Table 1. List of analyzed tissues from the GeneLab database.

Identifier	Title	Authors, title, publisher, version, DOI	Analyzed Tissue
OSD-48	Rodent Research-1 (RR1) NASA Validation Flight: Mouse liver transcriptomic, proteomic, and epigenomic data	Globus R, Galazka J, Marcu O, Saravia-Butler A, Fogle H, Bense N, Chakravarty K, Lai Polo S, Chen R, Boyko V, Gebre S, Costes S. "Rodent Research-1 (RR1) NASA Validation Flight: Mouse liver transcriptomic, proteomic, and epigenomic data", NASA Open Science Data Repository, Version 10, http://doi.org/10.26030/jq04-0n5	Liver
OSD-98	Rodent Research-1 (RR1) NASA Validation Flight: Mouse adrenal gland transcriptomic, proteomic, and epigenomic data	Galazka J, Globus R. "Rodent Research-1 (RR1) NASA Validation Flight: Mouse adrenal gland transcriptomic, proteomic, and epigenomic data", NASA Open Science Data Repository, Version 7, http://doi.org/10.26030/n1jq-2364	Adrenal gland
OSD-99	Rodent Research-1 (RR1) NASA Validation Flight: Mouse extensor digitorum longus muscle transcriptomic and epigenomic data	Galazka J, Globus R. "Rodent Research-1 (RR1) NASA Validation Flight: Mouse extensor digitorum longus muscle transcriptomic and epigenomic data", NASA Open Science Data Repository, Version 4, http://doi.org/10.26030/1h3m-3q49	Extensor digitorum longus muscle
OSD-100	Rodent Research-1 (RR1) NASA Validation Flight: Mouse eye transcriptomic and epigenomic data	Globus R, Galazka J. "Rodent Research-1 (RR1) NASA Validation Flight: Mouse eye transcriptomic and epigenomic data", NASA Open Science Data Repository, Version 4, http://doi.org/10.26030/whek-4p98	Eye
OSD-101	Rodent Research-1 (RR1) NASA Validation Flight: Mouse left gastrocnemius muscle transcriptomic, proteomic, and epigenomic data	Galazka J, Globus R. "Rodent Research-1 (RR1) NASA Validation Flight: Mouse left gastrocnemius muscle transcriptomic, proteomic, and epigenomic data", NASA Open Science Data Repository, Version 5, http://doi.org/10.26030/sdmt-ae51	Gastrocnemius muscle
OSD-102	Rodent Research-1 (RR1) NASA Validation Flight: Mouse kidney transcriptomic, proteomic, and epigenomic data	Galazka J, Globus R. "Rodent Research-1 (RR1) NASA Validation Flight: Mouse kidney transcriptomic, proteomic, and epigenomic data", NASA Open Science Data Repository, Version 4, http://doi.org/10.26030/yn9m-2d19	Kidney
OSD-103	Rodent Research-1 (RR1) NASA Validation Flight: Mouse quadriceps muscle transcriptomic, proteomic, and epigenomic data	Galazka J, Globus R. "Rodent Research-1 (RR1) NASA Validation Flight: Mouse quadriceps muscle transcriptomic, proteomic, and epigenomic data", NASA Open Science Data Repository, Version 4, http://doi.org/10.26030/9vzk-b116	Quadriceps muscle

Identifier	Title	Authors, title, publisher, version, DOI	Analyzed Tissue
OSD-104	Rodent Research-1 (RR1) NASA Validation Flight: Mouse soleus muscle transcriptomic and epigenomic data	Galazka J, Globus R. "Rodent Research-1 (RR1) NASA Validation Flight: Mouse soleus muscle transcriptomic and epigenomic data", NASA Open Science Data Repository, Version 4, http://doi.org/10.26030/em9r-w619	Soleus muscle
OSD-105	Rodent Research-1 (RR1) NASA Validation Flight: Mouse tibialis anterior muscle transcriptomic, proteomic, and epigenomic data	Galazka J, Globus R. "Rodent Research-1 (RR1) NASA Validation Flight: Mouse tibialis anterior muscle transcriptomic, proteomic, and epigenomic data", NASA Open Science Data Repository, Version 4, http://doi.org/10.26030/xgw6-6t64	Tibialis anterior muscle
OSD-168	RR-1 and RR-3 mouse liver transcriptomics with and without ERCC control RNA spike-ins	Galazka J. "RR-1 and RR-3 mouse liver transcriptomics with and without ERCC control RNA spike-ins", NASA Open Science Data Repository, Version 10, http://doi.org/10.26030/rwyp-9325	Liver
OSD-238	Transcriptomic analysis of dorsal skin from mice flown on the MHU-2 mission	Ohno H, Galazka J, Lai Polo S, Saravia-Butler A, Fogle H, Boyko V, Dinh M, Costes S, Gebre S. "Transcriptomic analysis of dorsal skin from mice flown on the MHU-2 mission", NASA Open Science Data Repository, Version 7, http://doi.org/10.26030/cdv4-tn30	Dorsal skin
OSD-239	Transcriptomic analysis of femoral skin from mice flown on the MHU-2 mission	Ohno H, Galazka J, Lai Polo S, Saravia-Butler A, Fogle H, Boyko V, Dinh M, Costes S, Gebre S. "Transcriptomic analysis of femoral skin from mice flown on the MHU-2 mission", NASA Open Science Data Repository, Version 7, http://doi.org/10.26030/s7k9-7958	Femoral skin
OSD-240	Transcriptional analysis of dorsal skin from mice flown on the RR-5 mission	Galazka J, Soo C, Lai Polo S, Saravia-Butler A, Fogle H, Bense N, Boyko V, Dinh M, Costes S, Gebre S. "Transcriptional analysis of dorsal skin from mice flown on the RR-5 mission", NASA Open Science Data Repository, Version 8, http://doi.org/10.26030/6eg2-wz66	Dorsal skin
OSD-241	Transcriptional analysis of femoral skin from mice flown on the RR-5 mission	Galazka J, Soo C, Lai Polo S, Saravia-Butler A, Fogle H, Bense N, Boyko V, Dinh M, Costes S, Gebre S. "Transcriptional analysis of femoral skin from mice flown on the RR-5 mission", NASA Open Science Data Repository, Version 8, http://doi.org/10.26030/gfsz-c144	Femoral skin

Identifier	Title	Authors, title, publisher, version, DOI	Analyzed Tissue
OSD-254	Transcriptional analysis of dorsal skin from mice flown on the RR-7 mission	Galazka J, Lai Polo S, Saravia-Butler A, Fogle H, Bense N, Boyko V, Chen Y, Costes S, Gebre S. "Transcriptional analysis of dorsal skin from mice flown on the RR-7 mission", NASA Open Science Data Repository, Version 13, http://doi.org/10.26030/dcq8-6c70	Dorsal skin
OSD-530	Cell-free RNA analysis of plasma samples collected from six astronauts in JAXA Cell-Free Epigenome (CFE) Study	Muratani M. "Cell-free RNA analysis of plasma samples collected from six astronauts in JAXA Cell-Free Epigenome (CFE) Study", NASA Open Science Data Repository, Version 1, http://doi.org/10.26030/r2xr-h714	Plasma

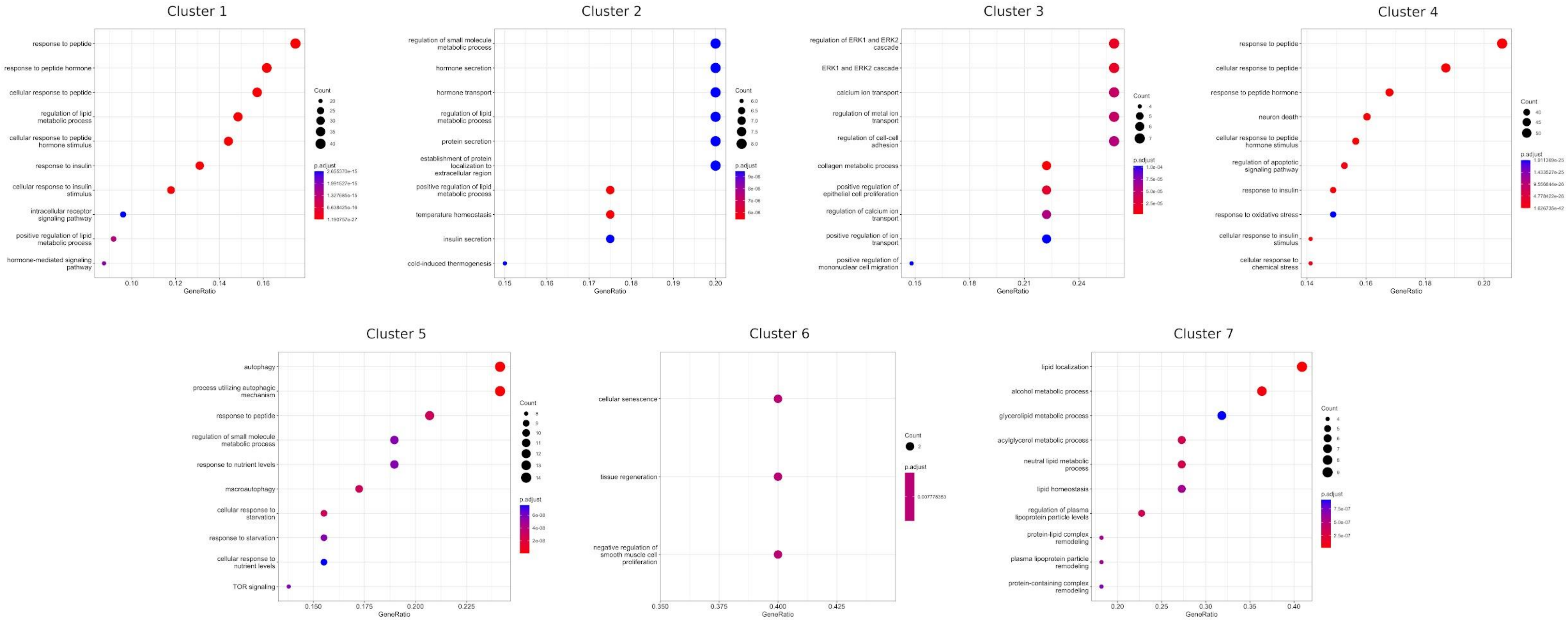

Supplementary Figure 1. Enriched GO processes for each of the seven clusters related to insulin signaling and insulin resistance from **Fig 1D**.
