# Supplementary material for: Spaceflight induces changes in gene expression profiles linked to insulin and estrogen
Source: Commun Biol. 2024 Jun 11;7:692. doi: 10.1038/s42003-023-05213-2 (PMC11166981; doi:10.1038/s42003-023-05213-2)
Supplement: Supplementary file 3 — Description of Additional Supplementary Files [file 42003_2023_5213_MOESM3_ESM.pdf]

Description of Additional Supplementary Files

File name: Supplementary Data 1
Description: Significant genes in insulin signaling between EDL and Soleus muscles.
