# Supplementary material for: Spaceflight induces changes in gene expression profiles linked to insulin and estrogen
Source: Commun Biol. 2024 Jun 11;7:692. doi: 10.1038/s42003-023-05213-2 (PMC11166981; doi:10.1038/s42003-023-05213-2)
Supplement: Supplementary file 5 — Reporting Summary [file 42003_2023_5213_MOESM5_ESM.pdf]

nature portfolio

Corresponding author(s): Afshin Beheshti

Last updated by author(s): Jul 31, 2023

Reporting Summary

Nature Portfolio wishes to improve the reproducibility of the work that we publish. This form provides structure for consistency and transparency in reporting. For further information on Nature Portfolio policies, see our Editorial Policies and the Editorial Policy Checklist.

Statistics

For all statistical analyses, confirm that the following items are present in the figure legend, table legend, main text, or Methods section.

n/a	Confirmed	
☐	☒	The exact sample size (*n*) for each experimental group/condition, given as a discrete number and unit of measurement
☒	☐	A statement on whether measurements were taken from distinct samples or whether the same sample was measured repeatedly
☐	☒	The statistical test(s) used AND whether they are one- or two-sided<br>*Only common tests should be described solely by name; describe more complex techniques in the Methods section.*
☒	☐	A description of all covariates tested
☒	☐	A description of any assumptions or corrections, such as tests of normality and adjustment for multiple comparisons
☐	☒	A full description of the statistical parameters including central tendency (e.g. means) or other basic estimates (e.g. regression coefficient) AND variation (e.g. standard deviation) or associated estimates of uncertainty (e.g. confidence intervals)
☐	☒	For null hypothesis testing, the test statistic (e.g. *F*, *t*, *r*) with confidence intervals, effect sizes, degrees of freedom and *P* value noted<br>*Give P values as exact values whenever suitable.*
☒	☐	For Bayesian analysis, information on the choice of priors and Markov chain Monte Carlo settings
☒	☐	For hierarchical and complex designs, identification of the appropriate level for tests and full reporting of outcomes
☒	☐	Estimates of effect sizes (e.g. Cohen's *d*, Pearson's *r*), indicating how they were calculated

Our web collection on statistics for biologists contains articles on many of the points above.

Software and code

Policy information about availability of computer code

Data collection	Majority of the datasets utilized are publicly available at the NASA GeneLab website (https://osdr.nasa.gov/bio/repo), including rodent data; Adrenal glands (OSD-98), Extensor digitorum longus muscle (OSD-99), Eye (OSD-100), Gastrocnemius muscle (OSD-101), Kidney (OSD-102), Quadriceps Muscle (OSD-103), Soleus muscle (OSD-104), Tibialis anterior muscle (OSD-105), Liver (OSD-168, OSD-48), Skin (OSD-238, OSD-239, OSD-240, OSD-241, OSD-254), JAXA CFE data (OSD-530).
Data analysis	All omics data related to mouse samples were obtained from NASA's GeneLab public omics repository. Heatmaps were made for the specific genes on the normalized values per time point using R package pheatmap version 1.0.12 and ComplexHeatmap R package. . The plots of normalized enrichment score (NES) of the different pathways for a given dataset are rendered using ggplot2

For manuscripts utilizing custom algorithms or software that are central to the research but not yet described in published literature, software must be made available to editors and reviewers. We strongly encourage code deposition in a community repository (e.g. GitHub). See the Nature Portfolio guidelines for submitting code & software for further information.

Data

Policy information about availability of data

All manuscripts must include a data availability statement. This statement should provide the following information, where applicable:

- Accession codes, unique identifiers, or web links for publicly available datasets
- A description of any restrictions on data availability
- For clinical datasets or third party data, please ensure that the statement adheres to our policy

Majority of the datasets utilized are publicly available at the NASA GeneLab website (https://osdr.nasa.gov/bio/repo), including rodent data; Adrenal glands (OSD-98), Extensor digitorum longus muscle (OSD-99), Eye (OSD-100), Gastrocnemius muscle (OSD-101), Kidney (OSD-102), Quadriceps Muscle (OSD-103), Soleus muscle (OSD-104), Tibialis anterior muscle (OSD-105), Liver (OSD-168, OSD-48), Skin (OSD-238, OSD-239, OSD-240, OSD-241, OSD-254), JAXA CFE data (OSD-530).

Research involving human participants, their data, or biological material

Policy information about studies with human participants or human data. See also policy information about sex, gender (identity/presentation), and sexual orientation and race, ethnicity and racism.

Reporting on sex and gender	For the human astronaut data in the paper we performed a sex independent analysis.
Reporting on race, ethnicity, or other socially relevant groupings	This was not reported in the manuscript.
Population characteristics	This was not reported in the manuscript. Only time in spaceflight was reported.
Recruitment	No bias in recruitment and this does not apply since we studied the limited astronaut data available to us.
Ethics oversight	Weill Cornell Medicine had the IRB for the Inspiration4 data. The JAXA data was obtained from public repository on GeneLab.

Note that full information on the approval of the study protocol must also be provided in the manuscript.

Field-specific reporting

Please select the one below that is the best fit for your research. If you are not sure, read the appropriate sections before making your selection.

☒ Life sciences ☐ Behavioural & social sciences ☐ Ecological, evolutionary & environmental sciences

For a reference copy of the document with all sections, see nature.com/documents/nr-reporting-summary-flat.pdf

Life sciences study design

All studies must disclose on these points even when the disclosure is negative.

Sample size	No sample size calucation was done. The data was obtained from previously performed experiments from GeneLab and we did not design the experiments.
Data exclusions	No data was excluded from the analysis.
Replication	Data was obtained from GeneLab. All biological replicates were used. The replication is difficult to do other than what is available since flying samples to space is limited.
Randomization	Randomization cannot be done. There are two sets of experimental conditions: 1) samples on the ground (i.e. Earth) and 2) samples flown to space (i.e. International Space Station or in orbit). The samples are then analyzed accordingly.
Blinding	Blinding was not possible, since we obtained the data from the GeneLab and there only exists two conditions per dataset.

Reporting for specific materials, systems and methods

We require information from authors about some types of materials, experimental systems and methods used in many studies. Here, indicate whether each material, system or method listed is relevant to your study. If you are not sure if a list item applies to your research, read the appropriate section before selecting a response.

Materials & experimental systems

n/a	Involved in the study
☒	☐ Antibodies
☒	☐ Eukaryotic cell lines
☒	☐ Palaeontology and archaeology
☐	☒ Animals and other organisms
☒	☐ Clinical data
☒	☐ Dual use research of concern
☒	☐ Plants

Methods

n/a	Involved in the study
☒	☐ ChIP-seq
☒	☐ Flow cytometry
☒	☐ MRI-based neuroimaging

Animals and other research organisms

Policy information about studies involving animals; ARRIVE guidelines recommended for reporting animal research, and Sex and Gender in Research

Laboratory animals	All experiments and data was obtained from the public database, GeneLab. We did not perform these experiments. That said the mice used were forty 12-week-old C57Bl/6J (Jackson Lab, Bar Harbor, ME) were selected based on similar body weights for 4 experimental groups (16-week-old at launch, n=10/group): baseline control, vivarium control, habitat control, and flight. Flight mice were launched onboard the ISS on September 21, 2014.
Wild animals	For the RR-1 mission, in summary, forty 12-week-old C57Bl/6J (Jackson Lab, Bar Harbor, ME) were selected based on similar body weights for 4 experimental groups (16-week-old at launch, n=10/group): baseline control, vivarium control, habitat control, and flight. Flight mice were launched onboard the ISS on September 21, 2014. Relevant to this study, the habitat control was placed in the ISS Environmental Simulator at NASA KSC on a 4-day delay to mimic flight temperature, CO2, and humidity conditions on the ISS for the duration of spaceflight. Flight mice were exposed to microgravity for a total of 37 days (33 days on ISS and 4 days in the Dragon Capsule). Mice were euthanized by injection of Euthasol followed by cervical dislocation and immediately fast frozen intact or partially dissected prior to carcasses being frozen in the Minus Eighty Degree Laboratory Freezer (MELFI) aboard the ISS. All ground mice were processed similarly. Flight mice carcasses returned to Earth February 2015 and were then maintained along with the control mice frozen at the Biospecimen Sharing Program (BSP) at the Ames Research Center until dissection.
Reporting on sex	Female mice were used only
Field-collected samples	. Relevant to this study, the habitat control was placed in the ISS Environmental Simulator at NASA KSC on a 4-day delay to mimic flight temperature, CO2, and humidity conditions on the ISS for the duration of spaceflight. Flight mice were exposed to microgravity for a total of 37 days (33 days on ISS and 4 days in the Dragon Capsule).
Ethics oversight	The data was obtained from previous experiments. The ethics were done according to the original study and material is provided on the GeneLab website for this.

Note that full information on the approval of the study protocol must also be provided in the manuscript.
